# Bronchial epithelial gene expression and interstitial lung abnormalities

**DOI:** 10.1186/s12931-023-02536-w

**Published:** 2023-10-10

**Authors:** Aravind A. Menon, Minyi Lee, Xu Ke, Rachel K. Putman, Takuya Hino, Jonathan A. Rose, Fenghai Duan, Samuel Y. Ash, Michael H. Cho, George T. O’Connor, Josée Dupuis, Hiroto Hatabu, Marc E. Lenburg, Ehab S. Billatos, Gary M. Hunninghake, the DECAMP Investigators, Avi Spira, Elizabeth Moses, Jennifer Beane, Josh Campbell, Jack Cunningham, Gang Liu, Hanqiao Liu, Sherry Zhang, Jiarui Zhang, Xingyi Shi, Carter Merenstein, Yue Zhao, Denise Aberle, Mitchell Schnall, Charles Apgar, Irene Mahon, Lindsey Dymond, Joe Bauza, Sarah Gevo, Constantine Gastonis, Helga Marquez, David Elashoff, Ignacio Wistuba, Humam Kadara, Junya Fujimoto, Clifton Dalgard, Matthew Wilkerson, Denise Aberle, George Washko, Charles M. Kinsey, Denise Fine, Ron Goldstein, Kathleen LaCerda, John Battaile, Mitchell Kroll, Bob Keith, Mary Jackson, Steve Dubinett, Gina Lee, Babak Aryanfar, Rafael Corona, Anil Vachani, Sam Soloman, Charles Atwood, Gregory Owens, Hanna Edvardsson, Pierre Massion, Trey Helton, Mary Reid, Chris Kuzniewski, Jacob Carmichael, Holly LaPerriere, J. ScottParrish, Lindsey White, Anna Kaur, Robert Browning, Maggie Nelissery, Folashade Akanni, Luis Rojas

**Affiliations:** 1 Division of Pulmonary and Critical Care Medicine, Brigham and Women’s Hospital, Harvard Medical School, 75 Francis Street, Boston, MA 02115 USA; 2 Section of Computational Biomedicine, Boston University School of Medicine, Boston, MA USA; 3 Department of Radiology, Brigham and Women’s Hospital, Harvard Medical School, Boston, MA USA; 4 Department of Biostatistics and Center for Statistical Sciences, Brown University School of Public Health, Providence, RI USA; 5 Pulmonary Center, Boston University School of Medicine, Boston, MA USA; 6 Channing Division of Network Medicine, Brigham and Women’s Hospital, Boston, MA USA; 7 Department of Biostatistics, Boston University School of Public Health, Boston, MA USA

**Keywords:** Interstitial lung abnormalities, ILA, Gene expression, Interstitial lung disease, Lung fibrosis

## Abstract

**Introduction:**

Interstitial lung abnormalities (ILA) often represent early fibrotic changes that can portend a progressive fibrotic phenotype. In particular, the fibrotic subtype of ILA is associated with increased mortality and rapid decline in lung function. Understanding the differential gene expression that occurs in the lungs of participants with fibrotic ILA may provide insight into development of a useful biomarker for early detection and therapeutic targets for progressive pulmonary fibrosis.

**Methods:**

Measures of ILA and gene expression data were available in 213 participants in the Detection of Early Lung Cancer Among Military Personnel (DECAMP1 and DECAMP2) cohorts. ILA was defined using Fleischner Society guidelines and determined by sequential reading of computed tomography (CT) scans. Primary analysis focused on comparing gene expression in ILA with usual interstitial pneumonia (UIP) pattern with those with no ILA.

**Results:**

ILA was present in 51 (24%) participants, of which 16 (7%) were subtyped as ILA with a UIP pattern. One gene, pro platelet basic protein (PPBP) and seventeen pathways (e.g. TNF-α signalling) were significantly differentially expressed between those with a probable or definite UIP pattern of ILA compared to those without ILA. 16 of these 17 pathways, but no individual gene, met significance when comparing those with ILA to those without ILA.

**Conclusion:**

Our study demonstrates that abnormal inflammatory processes are apparent in the bronchial airway gene expression profiles of smokers with and without lung cancer with ILA. Future studies with larger and more diverse populations will be needed to confirm these findings.

**Supplementary Information:**

The online version contains supplementary material available at 10.1186/s12931-023-02536-w.

## Introduction

Interstitial lung abnormalities (ILA) are chest computed tomography (CT) findings in persons not known to have interstitial lung disease (ILD) that may represent an early stage of pulmonary fibrosis (PF) [1]. Overlapping associations with genetic polymorphisms [2] and protein biomarkers [3] between those with ILA and idiopathic pulmonary fibrosis (IPF) highlight the fact that these conditions may share pathobiology.

While there are numerous cell types in the lung that likely play important roles in the pathobiology of IPF, growing data suggests that patients with PF have altered patterns of bronchial epithelial cell gene expression [4]. Significant alterations in airway morphology and gene expression signatures have been described in patients with IPF [5–9]. Additionally, several of the single nucleotide polymorphisms that are associated with increased risk for IPF, including MUC5B and DSP are expressed in bronchial airway cells [10, 11]. However, no prior study has characterized bronchial epithelial gene expression among persons with ILA.

To provide an assessment of the genes and pathways associated with ILA, and ILA subtypes we performed bronchial airway epithelial gene expression analyses in patients enrolled in Detection of Early Lung Cancer Among Military Personnel (DECAMP 1 and DECAMP 2) cohorts [5].

## Methods

### Study population

The DECAMP Study is a multicenter consortium comprised of 15 military treatment facilities, Veterans Affairs hospitals, and academic centers across the United States. Participants were recruited into one of two study protocols, designated as DECAMP-1 and DECAMP-2 [12]. Studies were registered at clinicaltrials.gov as DECAMP 1 (NCT01785342) and DECAMP 2 (NCT02504697) respectively. Briefly, study participants of DECAMP-1 were adults aged 45 and older with indeterminate pulmonary nodules and heavy smoking history whereas, study participants of DECAMP-2 were aged 50–79 with a heavy smoking history and a family history of lung cancer or a personal history of chronic obstructive pulmonary disease (COPD). This study was approved by the Human Research Protection Office (HRPO) for the Department of Defense, and the individual site IRBs for every participating site. All subjects were approached for written informed consent to participate in the study per IRB regulations (Additional file 1: Table S1).

### Biospecimen collection in DECAMP

All individuals in the DECAMP study underwent bronchoscopy. Bronchial airway epithelial cells were obtained from brushings of the right mainstem bronchus collected during fiberoptic bronchoscopy with an endoscopic cytobrush (Cellebrity Endoscopic Cytology Brush, Boston Scientific, Boston). The brushes were immediately placed in 1 mL of RNAprotect Cell Reagent (Qiagen, Valencia, CA) and kept at − 80 °C until RNA isolation was performed.

### RNA isolation, sequencing and data pre-processing

Total RNA was isolated using the miRNeasy Mini Kit (Qiagen, Valencia, CA). RNA integrity was assessed by Agilent BioAnalyzer, and RNA purity confirmed using a NanoDrop spectrophotometer. Libraries were generated using the Illumina TruSeq Stranded Total RNA kit and sequenced on the Illumina NextSeq 500 and Illumina HiSeq3000 with 75 base-pair paired-end reads (Illumina, San Diego, CA). For data preprocessing, we developed an automatic pipeline using the Nextflow framework. Quality of FASTQ files was assessed with FastQC. Reads were aligned to the human genome with 2-pass STAR and gene-level and isoform-level expression quantified with RSEM. Splice junction saturation, transcript integrity, and biotype distributions were calculated for each sample with RSeQC. DESeq2 or edgeR was used to identify associations between gene expression profiles and clinical variables while controlling for confounding covariates. Genetic variants were called using the Broad Institute’s GATK RNA-seq best-practices workflow. Briefly, duplicates were marked with Picard tools, splitting of intronic reads, realignment around indels, and base quality score recalibration were performed with GATK, and variants were called with Haplotypecaller.

### Imaging acquisition in DECAMP

DECAMP-1 utilized CT scans collected as part of routine clinical care while DECAMP-2 utilized a standardized protocol for image acquisition and reconstruction. DECAMP-2 scans were collected using low dose helical computed tomography on a minimum 16-slice scanner. The scans were acquired at 2.5 to 5 mm but reconstructed into 1 mm slice thickness using the soft tissue and lung algorithms. Images from all sites were then de-identified and submitted to the American College of Radiology Imaging Network (ACRIN) Core Laboratory for storage.

### Gene expression analysis

The LIMMA package in R (version 3.4.0) was used to assess differential gene expression (DGE). To do this, raw count matrix of gene expression was initially filtered by counts per million (CPM) such that a gene could only be included if its CPM was greater than 1 in 10% of the total number of patients. DGE analysis was then performed using a pairwise comparison between the ILA phenotypes at a false discovery rate (FDR) of 0.05. The differentially expressed genes (DEG) identified by LIMMA were further analyzed by Enrichr for over-representation analysis. Heatmaps were used to visualize the data and identify unsupervised gene clusters using the “Ward.D2” algorithm. Gene set enrichment analysis (GSEA) [13] was performed on pre-ranked gene lists created by pairwise comparisons between ILA status using Hallmark gene sets [14]. An FDR threshold of 0.01 was applied to select significant hallmark gene sets.

### ILA characterization

Measures of ILA were assessed on chest CT scans using a sequential method by readers (including radiologists and pulmonologists) blinded to prior interpretations and participant information and per Fleischner society recommendations [1]. Participants with indeterminate ILA status were excluded from this analysis (Additional file 2: Fig. S1) [15]. ILA with a UIP pattern was specifically characterised in those who had either a probable or definite UIP pattern as previously published [16].

### Statistical analysis

Data are presented as means and standard deviations for continuous measurements and number and percentage for categorical features. P values were calculated using Fisher’s exact test or t test as applicable. All analyses were adjusted for age, gender, cohort, cancer status, smoking status and sequencing method as confounders. Genes and pathways demonstrating a p-value < 0.05 after adjusting for the false discovery rate (FDR) were considered statistically significant.

## Results

Among the 213 participants with both ILA phenotyping and gene expression data, there were 51 (24%) with ILA and 162 (76%) without ILA (Additional file 2: Fig. S1). Of the 51 with ILA, 16 (30%) had a probable or definite UIP pattern (hereafter referred to as ‘ILA with a UIP pattern’) and 35 (70%) had ILA without a UIP pattern (hereafter referred to as ‘ILA with no UIP’). Compared to those without ILA and those with ILA with no UIP pattern, participants with a UIP pattern of ILA were older, and had increased measures of forced expiratory volume in one second (FEV1), forced vital capacity (FVC) and ratios of FEV1 to forced vital capacity (FEV1/FVC) (Table 1). Consistent with these results, participants with a UIP pattern of ILA had lower rates of COPD on spirometry (37.5% vs 71%; Fisher’s Exact Test; p = 0.009; Table 1). Those with ILA both without UIP pattern and with a UIP pattern had more malignant lung nodules compared to those without ILA (Table 1).

**Table 1:** Baseline characteristics of DECAMP participants comparing with ILA with a UIP pattern to those without ILA and ILA with no UIP variable

	No ILA (N = 162)	ILA with a UIP pattern^a^ (N = 16)	ILA with no UIP pattern^a^ (N = 35)	*P* value
Age (mean, SD)^a^	64 (8)	70 (6)	67 (6)	**0.004**
Sex				0.21
Male (n, %)	117 (72)	14 (88)	29 (83)	
Female (n, %)	45 (28)	2 (12)	6 (17)	
Race				0.68
White (n, %)	120 (76)	13 (81)	26 (81)	
Black (n, %)	27 (17)	1 (6)	4 (13)	
Asian (n, %)	5 (3)	1 (6)	0 (0)	
Smoking status				0.85
Current (n, %)	74 (46)	7 (44)	14 (40)	
Former (n, %)	81 (50)	9 (56)	20 (57)	
Unknown (n, %)	7 (4)	0 (0)	1 (2.9)	
Spirometry				
FEV1% predicted (mean, SD)^a^	72.0 (20)	93.0 (13)	75.7 (17)	**< 0.001**
FEV1/FVC (mean, SD)	0.6 (0.1)	0.7 (0.1)	0.7 (0.1)	**0.004**
FVC % predicted (mean, SD)^a^	87.6 (18)	99.1 (15)	85.0 (15)	**0.018**
COPD^b^	116 (71.6)	6 (37.5)	19 (54.3)	**0.014**
Radiology^c^				
Emphysema (mean, SD) (% of lung volume < 950HU)^a^	5.4 (7.7)	4.2 (3.4)	3.7 (5.9)	0.57
Cancer^d^				**< 0.001**
Yes (n, %)	13 (8)	8 (50)	7 (20)	
No (n, %)	21 (13)	6 (38)	4 (11)	
Sequencing method				0.42
Total (n, %)	141 (87)	12 (75)	30 (86)	
Exome (n, %)	21 (13)	4 (25)	5 (14)	

^a^*SD* standard deviation, *UIP* usual interstitial pneumonia, *FEV1* forced expiratory volume in 1st second, *FVC* forced vital capacity, *COPD* chronic obstructive pulmonary disease, *HU* Hounsfield units

^b^COPD was defined as FEV1/FVC < 0.7. Spirometry data was not available on 3 (2.5%) participants without ILA

^c^Radiographic data on emphysema was not available for 4 participants (25%) with ILA with a UIP pattern, 21 (60%) participants with ILA with no UIP and and 71 participants (44%) without ILA

^d^Cancer data was available only for DECAMP-1 participants. Additional details about cancer status is provided in Additional file 8: Table S7

After adjusting for multiple testing, no individual gene met statistical significance for differential expression between those with ILA (n = 51) compared to those without ILA (n = 162) (Additional file 2: Fig. S1 and Additional file 3: Table S2). Similarly no significant differential expression was observed when comparing ILA with no UIP (n = 35) and those without ILA (n = 162) (Additional file 4: Table S3).

**Fig. 1:**
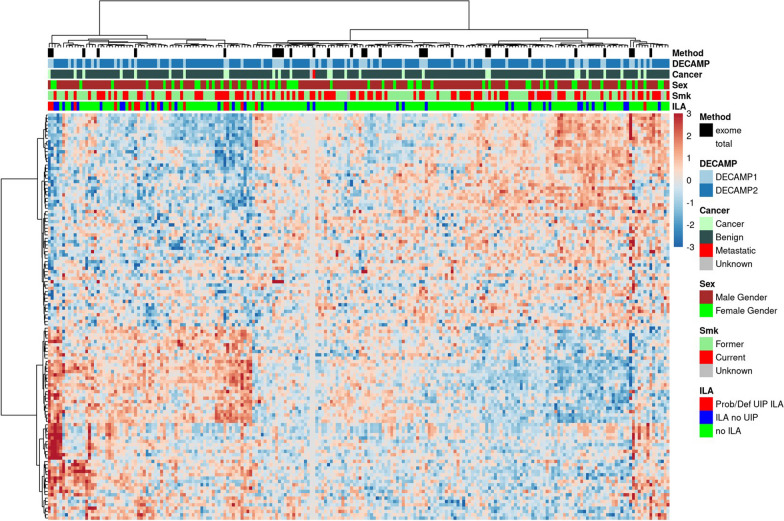
Heatmap of gene expression differences across study cohort

Participants with ILA with a UIP pattern compared to those without ILA, demonstrated a > eightfold upregulation of the gene encoding Pro-Platelet basic protein [PPBP] (FDR < 0.05). At a less stringent threshold, the 122 genes with p < 0.01 (Additional file 5: Table S4) divided the samples into two clusters including one that was enriched for samples from participants with ILA with a UIP pattern (Fisher’s test, p = 0.001; Fig. 1). We did not detect any other significant differences between the clusters with regard to the characteristics of the participants from whom the samples were obtained. The list of genes with UIP associated gene expression was highly overlapping when COPD was included as covariate (Fisher’s Exact Test; p < 1e−200).

Gene set enrichment analysis (GSEA) demonstrated seventeen pathways whose genes were significantly enriched among those with a UIP pattern of ILA when compared to those without ILA (FDR < 0.01) including tumour necrosis factor-alpha (TNF-α) signalling through nuclear factor kappa-B (NF-κB) (Table 2). 16 out of these 17 pathways were also amongst the 24 pathways significantly upregulated (FDR < 0.01) when comparing participants with ILA to that without ILA (Additional file 6: Table S5), a highly significant overlap (hypergeometric test; p < 7 × 10^–7^). GSEA of the ILA with no UIP group showed 13 pathways that met significance; of which 9 were common to the ones in the ILA with UIP group (hypergeometric test p = 0.003) and 12 in common with the whole ILA group (hypergeometric test; p = 0.0001) (Additional file 7: Table S6).

**Table 2:** Differentially expressed gene sets in patients with ILA in a probable or definite UIP pattern that met significance

Gene set	ES	P-value	FDR q-value
TNF-α signalling via NFKB	0.658	1.67E−20	8.35E−19
Inflammatory response	0.549	3.90E−10	9.74E−09
Heme metabolism	0.540	1.30E−08	2.16E−07
Interferon gamma response	0.482	1.75E−07	2.19E−06
Oxidative phosphorylation	0.493	2.39E−07	2.39E−06
MYC targets V1	0.471	5.29E−07	4.41E−06
Interferon alpha response	0.562	1.22E−06	8.69E−06
KRAS signalling up	0.468	5.10E−06	3.19E−05
Complement	0.450	1.92E−05	1.07E−04
E2F targets	0.438	2.25E−05	1.13E−04
MTORC1 signalling	0.430	4.22E−05	1.92E−04
IL6 JAK STAT3 signalling	0.550	1.03E−04	4.29E−04
IL2 STAT5 signalling	0.418	2.76E−04	1.06E−03
G2M checkpoint	0.405	3.48E−04	1.24E−03
Protein secretion	0.457	1.94E−03	6.06E−03
Cholesterol homeostasis	0.499	1.90E−03	6.06E−03
Apoptosis	0.391	2.16E−03	6.35E−03

*ES* enrichment score, *FDR* false discovery rate, *TNF* tumour necrosis factor, *NF-κB* nuclear factor Kappa-B, *MYC* master regulator of cell cycle entry and proliferative metabolism, *KRAS* Kirsten rat sarcoma viral gene oncogene homolog, *E2F* E2F family of transcription factors, *MTORC* mammalian target of rapamycin complex, *IL* interleukin, *JAK* Janus kinase, *STAT* signal transducer and activation of transcription, *G2M* G2-mitotic phase

## Discussion

This is the first assessment of bronchial epithelial gene expression among participants with ILA and these findings suggest that both the UIP pattern of ILA and ILA may be associated with similarly altered bronchial airway gene expression at the pathway level.

In this cohort, the most significantly differentially expressed gene was PPBP; a gene within the C-X-C motif cytokine family, specifically ligand 7. While CXCL7 has been associated with non-small cell lung cancer [17], it has also has been implicated in early stages of wound healing and has previously been associated with IPF, both in serum samples as well as bronchoalveolar lavage fluid [18, 19]. It is hypothesized that this cytokine helps to recruit mesenchymal stem cells from bone marrow and promote fibrosis [20].

Genes involved in TNF-α signalling were among the genes most induced in bronchial brushes from individuals with UIP pattern of ILA and in individuals with ILA relative to non-ILA controls. This pathway has similarly been demonstrated to be upregulated in the lungs of patients with pulmonary fibrosis [21], and it’s downregulation through the suppression of transforming growth factor-beta (TGF-β) is posited to be one of the therapeutic effects of pirfenidone [22]. These findings suggest that studying persons with ILA, can help to identify biologic pathways that are dysregulated in patients with clinically identified PF.

ILA with no UIP group may represent an intermediate step between the No ILA group and ILA with UIP group based on clinical characteristics and gene expression.

There are limitations to the present study. First, our ability to identify significant pathway enrichment among the genes most differentially expressed despite our overall failure to detect differential expression of individual genes suggests that our study is limited by its small sample size. Second, although analyses were adjusted for the presence of malignancy, it is difficult to exclude the possibility that residual differential background gene expression and the potential covariance of lung cancer and ILA could have affected our results. Third, we cannot rule out that performing this analysis on a subset of DECAMP, due to data availability influenced these results. Specifically, the prevalence of ILA in this cohort is larger than previously described and this may be due to selection bias from using a limited dataset of cohort participants with both ILA phenotyping and RNA sequencing data available. The results of the study are also limited to subjects with a known current or former smoking history and may differ from subjects with ILA without a smoking history. For these reasons, replication of these findings in additional cohorts will be important to validate the observed associations and assess generalizability to other populations.

## Conclusion

There are differentially expressed bronchial epithelial cell genes and pathways associated with ILA among patients in DECAMP. Some of the most differentially expressed genes and pathways have been similarly demonstrated to be upregulated among patients with IPF. Additional studies in larger cohorts are warranted to confirm these findings.

### Supplementary Information

**Additional file 1: Table S1.** Institutional Review Board committee names and project approval numbers for each center for the DECAMP Study.

**Additional file 2: Figure S1.** Study design diagram.

**Additional file 3: Table S2.** List of differentially expressed genes that met p-value < 0.01 among those with ILA as compared to those without ILA.

**Additional file 4: Table S3.** List of differentially expressed genes that met p < 0.01 among those without probable or definite UIP pattern of ILA as compared to those without ILA.

**Additional file 5: Table S4.** List of differentially expressed genes that met p-value < 0.01 among those with Probable or Definite UIP pattern of ILA as compared to those without ILA.

**Additional file 6: Table S5.** List of differentially expressed pathways comparing ILA with no ILA.

**Additional file 7: Table S6.** List of differentially expressed pathways comparing ILA no UIP with no ILA.

**Additional file 8: Table S7.** Cancer breakdown of nodule identified in participants in DECAMP 1 Cohort.

## Data Availability

The data that support the findings of this study are available from the DECAMP consortium but restrictions apply to the availability of these data, which were used under license for the current study, and so are not publicly available. Data are however available from the authors upon reasonable request and with permission of DECAMP consortium.

## Abbreviations

ILA: Interstitial lung abnormalities
DECAMP: Detection of Early Lung Cancer Among Military Personnel
CT: Computed tomography
UIP: Usual interstitial pneumonia
ILD: Interstitial lung disease
IPF: Idiopathic pulmonary fibrosis
PF: Pulmonary fibrosis
RNA: Ribonucleic acid
FDR: False discovery rate
PPBP: Pro-platelet basic protein
GSEA: Gene set enrichment analysis
TNF-α: Tumour necrosis factor-alpha
NF-κB: Nuclear factor kappa-B
CXCL7: C-X-C motif cytokine family, ligand 7
TGF-β: Transforming growth factor-beta

## References

[1] Hatabu H, Hunninghake GM, Richeldi L, Brown KK, Wells AU, Remy-Jardin M (2020). Interstitial lung abnormalities detected incidentally on CT: a position paper from the Fleischner Society. Lancet Respir Med.

[2] Hobbs BD, Putman RK, Araki T, Nishino M, Gudmundsson G, Gudnason V (2019). Overlap of genetic risk between interstitial lung abnormalities and idiopathic pulmonary fibrosis. Am J Respir Crit Care Med.

[3] Sanders JL, Putman RK, Dupuis J, Xu H, Murabito JM, Araki T (2021). The association of aging biomarkers, interstitial lung abnormalities, and mortality. Am J Respir Crit Care Med.

[4] Chakraborty A, Mastalerz M, Ansari M, Schiller HB, Staab-Weijnitz CA (2022). Emerging roles of airway epithelial cells in idiopathic pulmonary fibrosis. Cells.

[5] Ikezoe K, Hackett TL, Peterson S, Prins D, Hague CJ, Murphy D (2021). Small airway reduction and fibrosis is an early pathologic feature of idiopathic pulmonary fibrosis. Am J Respir Crit Care Med.

[6] Tanabe N, McDonough JE, Vasilescu DM, Ikezoe K, Verleden SE, Xu F (2020). Pathology of idiopathic pulmonary fibrosis assessed by a combination of microcomputed tomography, histology, and immunohistochemistry. Am J Pathol.

[7] Habermann AC, Gutierrez AJ, Bui LT, Yahn SL, Winters NI, Calvi CL (2020). Single-cell RNA sequencing reveals profibrotic roles of distinct epithelial and mesenchymal lineages in pulmonary fibrosis. Sci Adv.

[8] Prasse A, Binder H, Schupp JC, Kayser G, Bargagli E, Jaeger B (2019). BAL cell gene expression is indicative of outcome and airway basal cell involvement in idiopathic pulmonary fibrosis. Am J Respir Crit Care Med.

[9] De Sadeleer LJ, Verleden SE, Schupp JC, McDonough JE, Goos T, Yserbyt J (2022). BAL transcriptomes characterize idiopathic pulmonary fibrosis endotypes with prognostic impact. Chest.

[10] Noth I, Zhang Y, Ma SF, Flores C, Barber M, Huang Y (2013). Genetic variants associated with idiopathic pulmonary fibrosis susceptibility and mortality: a genome-wide association study. Lancet Respir Med.

[11] Hao Y, Bates S, Mou H, Yun JH, Pham B, Liu J (2020). Genome-wide association study: functional variant rs2076295 regulates desmoplakin expression in airway epithelial cells. Am J Respir Crit Care Med.

[12] Billatos E, Duan F, Moses E, Marques H, Mahon I, Dymond L (2019). Detection of early lung cancer among military personnel (DECAMP) consortium: study protocols. BMC Pulm Med.

[13] Subramanian A, Tamayo P, Mootha VK, Mukherjee S, Ebert BL, Gillette MA (2005). Gene set enrichment analysis: a knowledge-based approach for interpreting genome-wide expression profiles. Proc Natl Acad Sci USA.

[14] Liberzon A, Birger C, Thorvaldsdottir H, Ghandi M, Mesirov JP, Tamayo P (2015). The molecular signatures database (MSigDB) hallmark gene set collection. Cell Syst.

[15] Washko GR, Hunninghake GM, Fernandez IE, Nishino M, Okajima Y, Yamashiro T (2011). Lung volumes and emphysema in smokers with interstitial lung abnormalities. N Engl J Med.

[16] Putman RK, Gudmundsson G, Axelsson GT, Hida T, Honda O, Araki T (2019). Imaging patterns are associated with interstitial lung abnormality progression and mortality. Am J Respir Crit Care Med.

[17] Crossno PF, Polosukhin VV, Blackwell TS, Johnson JE, Markin C, Moore PE (2010). Identification of early interstitial lung disease in an individual with genetic variations in ABCA3 and SFTPC. Chest.

[18] Foster MW, Morrison LD, Todd JL, Snyder LD, Thompson JW, Soderblom EJ (2015). Quantitative proteomics of bronchoalveolar lavage fluid in idiopathic pulmonary fibrosis. J Proteome Res.

[19] Lee J, Arisi I, Puxeddu E, Mramba LK, Amicosante M, Swaisgood CM (2018). Bronchoalveolar lavage (BAL) cells in idiopathic pulmonary fibrosis express a complex pro-inflammatory, pro-repair, angiogenic activation pattern, likely associated with macrophage iron accumulation. PLoS ONE.

[20] Kalwitz G, Endres M, Neumann K, Skriner K, Ringe J, Sezer O (2009). Gene expression profile of adult human bone marrow-derived mesenchymal stem cells stimulated by the chemokine CXCL7. Int J Biochem Cell Biol.

[21] Frankel SK, Cosgrove GP, Cha SI, Cool CD, Wynes MW, Edelman BL (2006). TNF-alpha sensitizes normal and fibrotic human lung fibroblasts to Fas-induced apoptosis. Am J Respir Cell Mol Biol.

[22] Maher TM (2010). Pirfenidone in idiopathic pulmonary fibrosis. Drugs Today.

