# Supplementary material for: Bronchial epithelial gene expression and interstitial lung abnormalities
Source: Respir Res. 2023 Oct 10;24:245. doi: 10.1186/s12931-023-02536-w (PMC10566143; doi:10.1186/s12931-023-02536-w)
Supplement: Supplementary file 1 — Additional file 1: Table S1. Institutional Review Board committee names and project approval numbers for each center for the DECAMP Study. [file 12931_2023_2536_MOESM1_ESM.docx]

The DECAMP Study was approved by the individual site institutional review boards (IRB) at every participating site. In addition, the DECAMP study was approved by the Human Research Protection Office (HRPO) for the Department of Defense. All subjects were approached for written informed consent to participate in the study in accordance with IRB regulations. Further details regarding IRB committee names and project approval numbers for each center can be found below).

| **Site Name** | **ACRIN Site #** | **CTEP Inst #** | **FWA** | **FWA  Exp Date** | **DECAMP 1 IRB Approval** | **DECAMP 1 HRPO Log #** | **DECAMP 2  IRB Approval** | **DECAMP 2 HRPO Log #** |
| --- | --- | --- | --- | --- | --- | --- | --- | --- |
| University of Pennsylvania | 4202 | PA141 | FWA00004028 | 2/2/21 | Protocol #: 816341 | A-17242.1o | Protocol #: 818746 Review Board: IRB #2 | A-17242.2o |
| Brooke Army Medical Center | 4238 | TX055 | FWA00004092 | 12/5/21 | Project #: 376127 Reference #: C.2012.135 | A-17242.1m | Project #: 385999 Reference #: C.2013.107 | A-17242.2m |
| Roswell Park Memorial Institute | 4278 | NY158 | FWA00006731 | 5/4/23 | IRB ID: I 217812 | A-17242.1g | IRB ID: I 251914 | A-17242.2g |
| VA Greater Los Angeles Health Care System | 4438 | CA221 | FWA00000734 | 11/1/22 | VA Project #: 0051 | A-17242.1h | VA Project #: 0052 | A-17242.2h |
| UCLA | 4494 | CA006 | FWA00004642 | 6/22/23 | IRB#12-000926 | A-17242.1p | N/A | N/A |
| Philadelphia VA | 4714 | PA082 | FWA00001311 | 4/13/22 | ID: 01405 \| Prom #: 0002 | A-17242.1e | ID: 01428 \| Prom #: 0003 | A-17242.2e |
| VA Boston Healthcare System | 4790 | MA139 | FWA00001270 | 7/6/23 | IRB# 2661 | A-17242.1c | IRB# 2802 | A-17242.2c |
| VA North Texas Health Care System | 4791 | TX002 | FWA00001338 | 1/2/23 | IRB# 12-035 | A-17242.1d | IRB# 13-050 | A-17242.2d |
| VA Eastern Colorado Health Care System | 4792 | CO015 | FWA00005070 | 10/26/22 | COMIRB Protocol 12-0707 | A-17242.1f | COMIRB Protocol 12-1662 | A-17242.2f |
| Nashville VA Medical Center | 4793 | TN004 | FWA00003772 | 12/1/20 | Study ID#: 331929 | A-17242.1j | Study ID#: 470111 | A-17242.2j |
| VA Pittsburgh Healthcare System | 4794 | PA022 | FWA00001282 | 2/9/21 | Pro00000495 | A-17242.1i | Pro00000576 | A-17242.2i |
| Walter Reed National Military Medical Center | 4795 | MD001 | FWA00017749 | 9/9/21 | IRBnet #: 376221 | A-17242.1n | IRBnet #: 387954 | A-17242.2n |
| Naval Medical Center San Diego | 4796 | CA074 | FWA00002342 | 9/22/21 | Protocol CIP # NMCSD.2012.0025 | A-17242.1l | Protocol CIP # NMCSD.2013.0078 | A-17242.2l |
| Naval Medical Center Portsmouth | 4797 | VA024 | FWA00006001 | 11/23/21 | Protocol CIP # NMCP.2013.0006 | A-17242.1k | Protocol CIP # NMCP.2013.0046 | A-17242.2k |
| Boston Medical Center | 4798 | MA136 | FWA00000301 | 11/12/20 | IRB Number: H-31755 | A-17242.1a | IRB Number: H-32479 | A-17242.1a |
