# Supplementary material for: Bronchial epithelial gene expression and interstitial lung abnormalities
Source: Respir Res. 2023 Oct 10;24:245. doi: 10.1186/s12931-023-02536-w (PMC10566143; doi:10.1186/s12931-023-02536-w)

**Additional file 2 for Bronchial Epithelial Gene Expression and Interstitial Lung Abnormalities**

Aravind A. Menon, MD, MPH^1^*; Minyi Lee, BS^2*^; Xu Ke, MD, PhD ^2^,Rachel K. Putman, MD, MPH^1^; Takuya Hino MD^3^; Jonathan A. Rose MD,MS^1^; Fenghai Duan, PhD^4^; Samuel Y. Ash, MD, MPH^1^; Michael H. Cho MD, MPH^6^; George T. O’ Connor, MD, MS^5^; Josée Dupuis PhD^7^; Hiroto Hatabu, MD, PhD^3^; Marc E. Lenburg PhD^2^; Ehab S. Billatos, MD^5*^; Gary M. Hunninghake, MD, MPH^1*^ on behalf of the DECAMP Investigators^†^

^1^Division of Pulmonary and Critical Care Medicine, Brigham and Women’s Hospital, Boston, MA; ^2^Section of Computational Biomedicine, Boston University School of Medicine. Boston, MA; ^3^Department of Radiology, Brigham and Women’s Hospital, Harvard Medical School, Boston, MA; ^4^Department of Biostatistics and Center for Statistical Sciences, Brown University School of Public Health, Providence, RI; ^5^ Pulmonary Center, Boston University School of Medicine, Boston. MA, ^6^Channing Division of Network Medicine, Brigham and Women’s Hospital, Boston, MA, ^7^Department of Biostatistics, Boston University School of Public Health, Boston, MA

*AM and ML and ESB and GMH contributed equally to this work.

**Additional Results**

Figure S1: Study design diagram
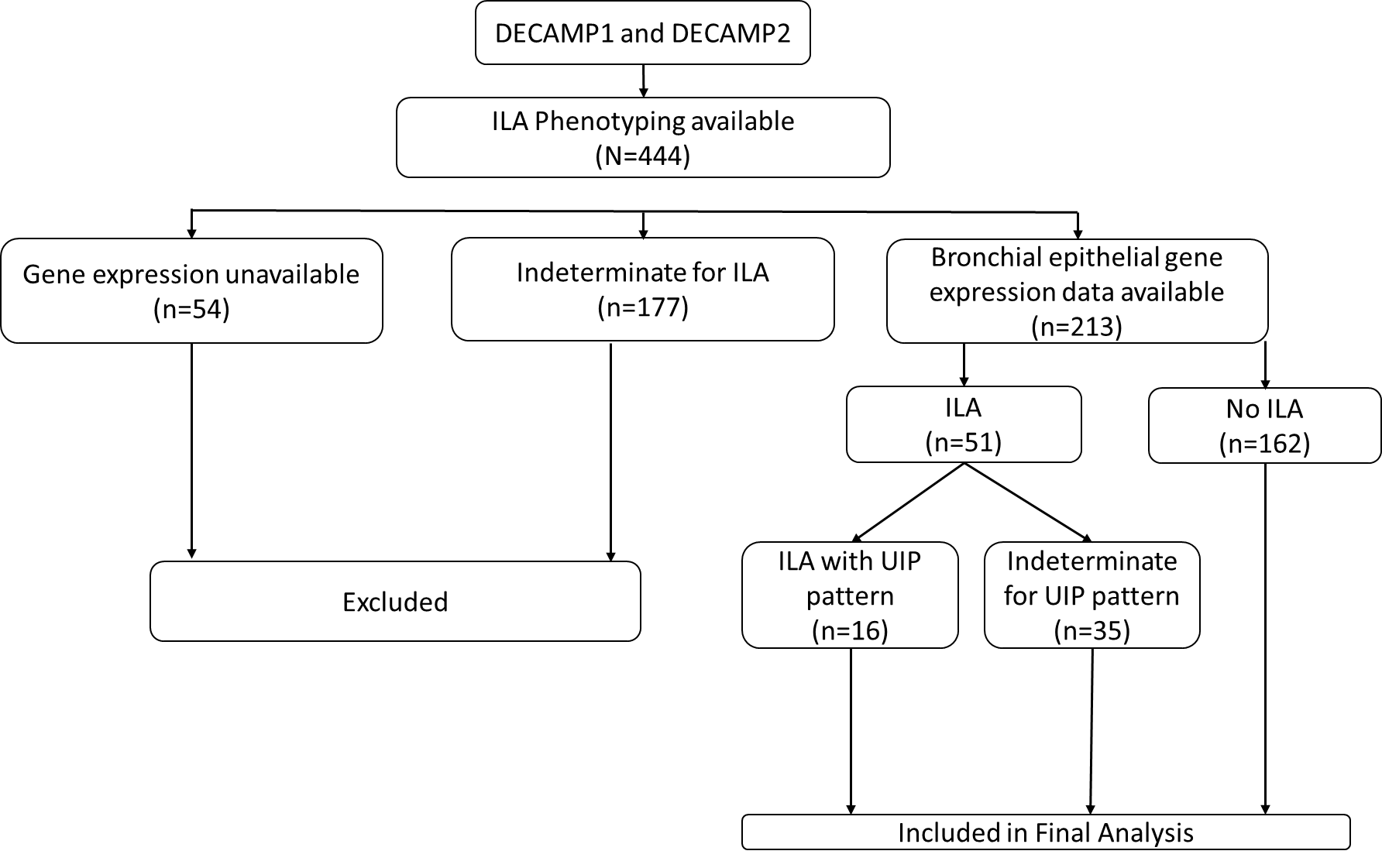

Supplement: Supplementary file 2 — Additional file 2: Figure S1. Study design diagram. [file 12931_2023_2536_MOESM2_ESM.docx]
