# Supplementary material for: Bronchial epithelial gene expression and interstitial lung abnormalities
Source: Respir Res. 2023 Oct 10;24:245. doi: 10.1186/s12931-023-02536-w (PMC10566143; doi:10.1186/s12931-023-02536-w)
Supplement: Supplementary file 4 — Additional file 4: Table S3. List of differentially expressed genes that met p < 0.01 among those without probable or definite UIP pattern of ILA as compared to those without ILA. [file 12931_2023_2536_MOESM4_ESM.docx]

Table S3: List of differentially expressed genes that met p < 0.01 among those without probable or definite UIP pattern of ILA as compared to those without ILA

| Gene ID | Gene Symbol | logFC | P.Value | adj.P.Val |
| --- | --- | --- | --- | --- |
| ENSG00000148426 | PROSER2 | -0.3400844 | 4.93E-05 | 0.85558435 |
| ENSG00000186523 | FAM86B1 | -0.3613507 | 0.00040805 | 0.99992721 |
| ENSG00000026751 | SLAMF7 | 0.65585185 | 0.00073067 | 0.99992721 |
| ENSG00000035681 | NSMAF | 0.17884269 | 0.00140008 | 0.99992721 |
| ENSG00000124588 | NQO2 | 0.23773775 | 0.00149203 | 0.99992721 |
| ENSG00000140416 | TPM1 | -0.2006494 | 0.00417283 | 0.99992721 |
| ENSG00000170298 | LGALS9B | -0.3643536 | 0.00476743 | 0.99992721 |
| ENSG00000183044 | ABAT | -0.1742241 | 0.00542141 | 0.99992721 |
| ENSG00000132196 | HSD17B7 | 0.1510993 | 0.00497854 | 0.99992721 |
| ENSG00000069849 | ATP1B3 | 0.21211313 | 0.00609363 | 0.99992721 |
| ENSG00000134152 | KATNBL1 | 0.2294921 | 0.00582936 | 0.99992721 |
| ENSG00000119900 | OGFRL1 | 0.23166145 | 0.00635613 | 0.99992721 |
| ENSG00000217128 | FNIP1 | 0.16052891 | 0.0065749 | 0.99992721 |
| ENSG00000132386 | SERPINF1 | -0.2390788 | 0.0066764 | 0.99992721 |
| ENSG00000132952 | USPL1 | 0.13018026 | 0.00644783 | 0.99992721 |
| ENSG00000158483 | FAM86C1 | -0.2454428 | 0.00295578 | 0.99992721 |
| ENSG00000185554 | NXF2 | -1.6309754 | 0.00364021 | 0.99992721 |
| ENSG00000197860 | SGTB | 0.43887043 | 0.00307717 | 0.99992721 |
| ENSG00000167207 | NOD2 | 0.30759353 | 0.00547573 | 0.99992721 |
| ENSG00000175029 | CTBP2 | -0.1267976 | 0.00922495 | 0.99992721 |
| ENSG00000175567 | UCP2 | -0.2282695 | 0.00959589 | 0.99992721 |
| ENSG00000006831 | ADIPOR2 | 0.11375333 | 0.00971416 | 0.99992721 |
| ENSG00000162645 | GBP2 | 0.48984407 | 0.00973099 | 0.99992721 |
| ENSG00000118777 | ABCG2 | 0.9662879 | 0.00114587 | 0.99992721 |
| ENSG00000167210 | LOXHD1 | -1.1572751 | 0.00481441 | 0.99992721 |
| ENSG00000187626 | ZKSCAN4 | 0.11482219 | 0.0094165 | 0.99992721 |
| ENSG00000158458 | NRG2 | -0.2817376 | 0.00641438 | 0.99992721 |
| ENSG00000143429 |  | -0.3911863 | 0.0025839 | 0.99992721 |
| ENSG00000251537 |  | -0.3775062 | 0.00744658 | 0.99992721 |
| ENSG00000152229 | PSTPIP2 | 0.31076276 | 0.00970073 | 0.99992721 |
| ENSG00000189046 | ALKBH2 | 0.15641054 | 0.00817509 | 0.99992721 |
| ENSG00000057704 | TMCC3 | 0.48096137 | 0.00954944 | 0.99992721 |
| ENSG00000186714 | CCDC73 | -0.2812381 | 0.00289415 | 0.99992721 |
| ENSG00000072694 | FCGR2B | 0.57897923 | 0.00844499 | 0.99992721 |
| ENSG00000267106 | C19orf82 | -0.1921263 | 0.0082968 | 0.99992721 |
| ENSG00000157322 | CLEC18A | -0.528395 | 0.00657135 | 0.99992721 |
| ENSG00000168269 | FOXI1 | -0.4854658 | 0.00614864 | 0.99992721 |
| ENSG00000185291 | IL3RA | 0.64525669 | 0.00551666 | 0.99992721 |
| ENSG00000185565 | LSAMP | 0.70484882 | 0.00311879 | 0.99992721 |
| ENSG00000206535 | LNP1 | -0.2014102 | 0.0059609 | 0.99992721 |
| ENSG00000099994 | SUSD2 | -0.6436525 | 0.0011373 | 0.99992721 |
| ENSG00000184515 | BEX5 | 0.50835924 | 0.00559166 | 0.99992721 |
| ENSG00000152766 | ANKRD22 | 0.60165885 | 0.00608517 | 0.99992721 |
| ENSG00000184933 | OR6A2 | -0.4423196 | 0.00354773 | 0.99992721 |
| ENSG00000171094 | ALK | -0.4004495 | 0.00490894 | 0.99992721 |
| ENSG00000144681 | STAC | 0.76424156 | 0.00415503 | 0.99992721 |
| ENSG00000147166 | ITGB1BP2 | -0.3584322 | 0.0061388 | 0.99992721 |
| ENSG00000131944 | C19orf40 | 0.28078939 | 0.00758352 | 0.99992721 |
| ENSG00000160326 | SLC2A6 | 0.62853554 | 0.00730586 | 0.99992721 |
| ENSG00000244457 | ENO1P1 | -1.2431839 | 0.009411 | 0.99992721 |
| ENSG00000168070 | C11orf85 | -0.5404686 | 0.00577545 | 0.99992721 |
| ENSG00000254712 |  | -0.5138278 | 0.00828141 | 0.99992721 |
| ENSG00000264727 |  | -0.7848325 | 0.00455891 | 0.99992721 |
| ENSG00000130032 | PRRG3 | 0.83175211 | 0.00981414 | 0.99992721 |
| ENSG00000270641 | TSIX | 0.8993748 | 0.00521542 | 0.99992721 |
| ENSG00000157103 | SLC6A1 | -1.0321686 | 0.0047828 | 0.99992721 |
| ENSG00000186487 | MYT1L | -1.1142185 | 0.00863565 | 0.99992721 |
